# Function and Regulation of Heterotrimeric G Proteins during Chemotaxis

**DOI:** 10.3390/ijms17010090

**Published:** 2016-01-14

**Authors:** Marjon E. Kamp, Youtao Liu, Arjan Kortholt

**Affiliations:** Department of Cell Biochemistry, University of Groningen, Nijenborgh 7, 9747 AG Groningen, The Netherlands; m.e.kamp@rug.nl (M.E.K.); youtao.liu@rug.nl (Y.L.)

**Keywords:** chemotaxis, G-protein coupled receptors, heterotrimeric G proteins, adaptation, non-canonical regulators, Gα effectors

## Abstract

Chemotaxis, or directional movement towards an extracellular gradient of chemicals, is necessary for processes as diverse as finding nutrients, the immune response, metastasis and wound healing. Activation of G-protein coupled receptors (GPCRs) is at the very base of the chemotactic signaling pathway. Chemotaxis starts with binding of the chemoattractant to GPCRs at the cell-surface, which finally leads to major changes in the cytoskeleton and directional cell movement towards the chemoattractant. Many chemotaxis pathways that are directly regulated by Gβγ have been identified and studied extensively; however, whether Gα is just a handle that regulates the release of Gβγ or whether Gα has its own set of distinct chemotactic effectors, is only beginning to be understood. In this review, we will discuss the different levels of regulation in GPCR signaling and the downstream pathways that are essential for proper chemotaxis.

## 1. Introduction

Chemotaxis, the process of directed cell movement towards a chemical gradient, plays an important role in both prokaryotes and eukaryotes. Prokaryotic chemotaxis is essential for food scavenging, while in mammals, chemotaxis plays, for example, a role in wound healing and embryogenesis [1]. Defects in chemotaxis are critically linked to the progression of many diseases including cancer, asthma, atherosclerosis and other chronic inflammatory diseases [2]. Although cells can detect chemoattractant gradients of highly diverse chemical compounds produced by many different sources, the main signaling pathways regulating chemotaxis are highly conserved among eukaryotes [3].

The most commonly used model systems for studying chemotaxis are the slime mold *Dictyostelium discoideum* and mammalian neutrophils [4]. Although having clearly distinct physiological roles, *Dictyostelium* and neutrophils have a highly similar chemotactic behavior. They display strong chemotactic responses, their stimuli are well-defined and their chemotaxis is characterized by amoeboid migration, creating actin-rich pseudopods at the front and retracting the back of the cell using myosin filaments [3,5]. Chemotaxis is essential for the *Dictyostelium* life cycle: during the vegetative phase of their life cycle, *Dictyostelium* scavenges the soil for bacteria by chemotaxing towards folic acid released by bacteria; however, if food is scarce, *Dictyostelium* cells secrete cyclic AMP (cAMP), which is used as a chemoattractant by neighboring cells to form a multicellular structure with spores that can resist harsh conditions.

During its lifecycle, *Dictyostelium*, as well as neutrophils, have to cope with a wide range of chemoattractant concentrations, e.g., during development, *Dictyostelium* encounters cAMP gradients ranging from 3 nM to 10 µM [6,7]. Activation of G-protein coupled receptors (GPCRs) is at the very base of the signaling pathways that enable this very sensitive and broad chemotaxis response. Chemotaxis starts with binding of the chemoattractant to GPCRs at the cell surface. The receptors transmit these signals into the interior of the cell by activation and dissociation of the heterotrimeric G protein complex. This subsequently results in the activation of a complex network of signaling molecules and the coordinated remodelling of the cytoskeleton. The final outcome is cellular movement up the chemoattractant gradient [8,9].

In this review, we highlight the crucial role of regulators of GPCR and heterotrimeric G-protein signaling and discuss the heterotrimeric pathways regulating chemotaxis.

## 2. Regulation of GPCRs and Heterotrimeric G Proteins during Chemotaxis

### 2.1. Chemotaxis Receptors and Their Regulation

Cells are able to detect and respond to a wide variety of chemoattractants and repellents, including peptides, lipids, and small proteins of several classes [2]. Although the structure of these compounds is highly diverse, most of them are detected by receptors of the GPCR family. The human GPCR family consists of nearly 800 genes divided into three main families; β2 adrenergic–like receptors, glucagon-like receptors, and metabotropic neurotransmitter-like receptors [10]. Chemotaxis receptors belong to the family of β2 adrenergic-like receptors. An overview of the chemotaxis receptors discussed in this review, their respective ligands and their expression is provided in Table 1. GPCRs consist of seven transmembrane α-helices, with an intracellular C-terminus and an extracellular N-terminus [11]. The extracellular domain regulates accessibility of the receptor, the transmembrane is the main binding surface for the ligand and, through conformational changes, the signal is transduced to the intracellular domain, which interacts with and activates the heterotrimeric G protein signaling cascade (see below) [8]. To be able to detect both very low and high concentrations of chemoattractant and migrate in a complex environment of competing chemotaxis cues, GPCR activation is highly regulated.

**Table 1:** Overview of chemotaxis receptors discussed in this review, their respective ligands and expression profiles. NK cell: Natural Killer cell.

Receptor	Ligand(s)	Cellular Expression
CCR5	CCL2/3/4/5/13/15	T cell, NK cell, monocyte, macrophage, dendritic cell
CCR6	CCL19, β-defensin	B cell, T cell, NK cell, dendritic cell
CXCR2	CCL28, CXCL1/2/5/6/7/8	T cell, NK cell, neutrophil, monocyte, dendritic cell, granulocyte
CXCR4	CXCL12 (SDF-1)	B cell, T cell, NK cell neutrophil, monocyte, macrophage, dendritic cell, granulocyte, neurons
CXCR5	CXCL13	B cell, T cell
BLT1/2	LTB4	B cell, T cell, neutrophil, monocyte, macrophage, dendritic cell, granulocyte
LPA1	LPA	NK cell, macrophage
PAFR	PAF	B cell, neutrophil, monocyte
FPR1/2	Formyl peptides	T cell, neutrophil, monocyte, macrophage, dendritic cell
A_1_ receptor	Adenosine	Neutrophil, monocyte, macrophage, dendritic cell
A_2A_ receptor	Adenosine	B cell, NK cell, neutrophil, monocyte, macrophage, dendritic cell
A_2B_ receptor	Adenosine	B cell, NK cell, neutrophil, monocyte, macrophage, dendritic cell
A_3_ receptor	Adenosine	B cell, NK cell, neutrophil, monocyte, macrophage, dendritic cell
cAR1	cAMP	*Dictyostelium.* Peaks at 4 h of development, then drops dramatically, early aggregation
cAR2	cAMP	*Dictyostelium*. Peaks at 16 h of development, mound formation
cAR3	cAMP	*Dictyostelium*. Peaks at 4 h of development, then slowly decreases, late aggregation stage
cAR4	cAMP	*Dictyostelium*. Peaks at 20 h of development, culmination
To be identified	Folic acid	*Dictyostelium*. Vegetative cells

#### 2.1.1. Ligand Binding Properties and Expression

In *Dictyostelium*, four cAMP receptors (cAR) have been identified that are involved in chemotaxis (Table 1). To cope with an increase in extracellular cAMP concentration during the aggregation stage [12,13], cells express the cAR1-4 receptors sequentially and with decreasing affinities. cAR2-4 have a relatively low affinity for cAMP and are important during the multicellular stage, whereas cAR1 has a high affinity for cAMP and is essential for signal transduction during early development and chemotaxis. At the onset of *Dictyostelium* aggregation the very shallow (starting from 3 nM) cAMP gradient is detected by the high affinity (*K*_d_ of 30 nM) cAR1 receptor. At late aggregation stages, the cAMP concentrations increase, thereby saturating cAR1 receptors [14]. The cAR1 receptors become phosphorylated at this stage resulting in a five-fold lower affinity [15]. Subsequently, cAR1 expression is down-regulated, while expression of the low affinity cAR3 receptor (*K*_d_ of 100 nM), cAR2 and cAR4 (both *K*_d_ in the mM range) increases, thereby enabling the cell to respond to the higher concentrations of cAMP [16].

Neutrophils use a highly similar mechanism to sense adenosine released by tissue cells. Inflammation or injury of tissue cells results in a more than 100-fold increase in adenosine release [17]. The cells use a combination of low and high affinity receptors to cope with these different levels of adenosine: where A_1_ and A_3_ show an EC_50_ between 0.2–0.5 µM, A_2A_ an EC_50_ between 0.6–0.9 µM, and A_2B_ an EC_50_ between 16–64 µM for adenosine (Table 1) [18]. At low concentrations, both high affinity receptors A_1_ and A_3_ promote chemotaxis, while, at higher concentrations, the low affinity A_2_ receptors are activated and neutrophil recruitment is diminished [17].

#### 2.1.2. Receptor Adaptation and Internalization

Upon ligand binding, all GPCRs transiently induce their own phosphorylation (homologous desensitization) while, as exemplified below, several receptors in addition induce phosphorylation of other receptors (heterologous desensitization) [19]. The desensitization of the GPCRs is achieved by the uncoupling of heterotrimeric G proteins, making it impossible for the receptor to transduce the signal via Gα or Gβγ [20], whereas, upon removal of the ligand, resensitization is accomplished by fast recycling of the receptors and digestion of the ligand.

**Figure 1:**
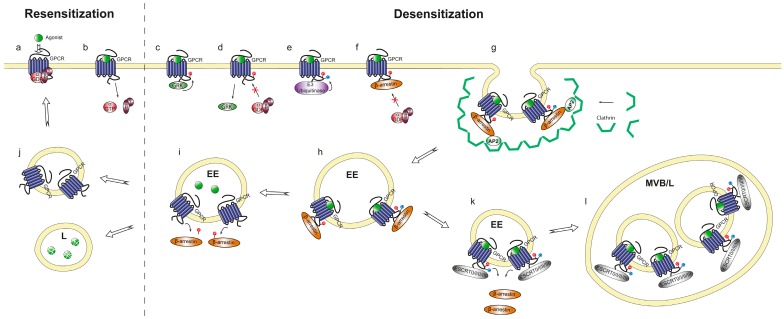
Overview of the different pathways of chemotaxis receptor adaptation and regulation. (**a**) Agonist binding; (**b**) Dissociation of heterotrimeric G proteins; (**c**) GPCR phosphorylation by GRK’s; (**d**) Reduced affinity for heterotrimeric G proteins due to phosphorylation; (**e**) Ubiquitination of the receptor, tagging it for the degradation pathway; (**f**) β-arrestin binds phosphorylated receptors and reduces receptor affinity for heterotrimeric G proteins; (**g**) Interaction of β-arrestin with β2 adaptin (AP2) and clathrin creates clathrin coated pits, essential for receptor internalization; (**h**) Endocytosis of receptors and agonists into early endosome (EE); (**i**) The pH in the endosomes drops, resulting in disassociation of the receptor and ligand; (**j**) Receptors are recycled to the membrane while the ligands are degraded in lysosomes (L); (**k**) In the degradation pathway, the ubiquitin tag is detected by the ESCRT proteins; (**l**) The ESCRT proteins target the receptor sequentially to the early endosomes, late endosomes, multivesicular bodies (MVB) and eventually to lysosomes where both the receptor and ligand are degraded.

The first step in desensitization is activation of G protein-coupled receptor kinases (GRKs), which subsequently phosphorylate the C-terminal domain of the receptors (Figure 1). Phosphorylated receptors not only have a decreased affinity for heterotrimeric G proteins, but more importantly, also bind with higher affinity to β-arrestins [21]. β-arrestin binding uncouples heterotrimeric G proteins from the receptor and can induce receptor internalization. Internalization can result in either recycling to the surface and resensitization, or degradation and persistent desensitization of receptors (Figure 1, [22,23]). Homologous receptor desensitization with associated changes in ligand affinities allows sensitivity to a broader concentration range of chemoattractant, which has been extensively studied for CXCR4, a chemokine receptor that responds to stromal derived factor 1 (SDF-1 or CXCL12). Upon CXCR4 phosphorylation, the E3 ubiquitin ligase IAP4 is recruited and ubiquitinates the C-terminal tail of CXCR4 (step 5 in Figure 1) [24,25]. The ubiquitin tags the receptor for lysosomal degradation through the endosomal sorting complex required for transport (ESCRT) pathway. The ubiquitin tag is detected by the ubiquitin binding domain (UBD) on the ESCRT proteins and transported to lysosomes where both the receptor and ligand are degraded (steps 11–12 in Figure 1) [26,27]. The receptor degradation reduces signaling in high concentration gradients, and stops cells from moving when they reach the source. Although the mechanism is not completely understood, homologous desensitization also seems to play a role in *Dictyostelium*, since a mutant strain expressing a non-phosphorylatable cAR1 showed impaired chemotaxis [28].

Neutrophils operate under very complex conditions of competing chemotaxis cues and opposing directions. Heterologous internalization allows classification of these signals, e.g., because of GRK-mediated receptor phosphorylation and degradation formyl peptides of bacterial or mitochondrial origin are dominant attractants over CXCL8 and LTB4 for neutrophil chemotaxis [29]. These properties are essential for neutrophil chemotaxis towards a necrotic core; they initially use the CXCL2 receptor (CXCR2) to migrate up an intravascular gradient of CXCL2, which they subsequently ignore and instead use FPR1 to migrate up a gradient of mitochondrion-derived formyl peptides.

Interestingly, recent studies have shown that β-arrestins not only regulate receptor adaptation, but also directly bind and activate downstream chemotaxis pathways. At the leading edge, β-arrestins can function as scaffold proteins for cofilin, which regulates actin polymerization at the front of the cell [30]. Furthermore, when the chemotaxis receptor CCR5 is activated by MIP1β (CCL4) a scaffold is made consisting of β-arrestin 2, PI3K and some non-receptor kinases. This β-arrestin 2 dependent scaffold is essential for MIP1β induced chemotaxis of human macrophages [31].

### 2.2. Kinetics and Regulation of Heterotrimeric G Proteins during Chemotaxis

**Figure 2:**
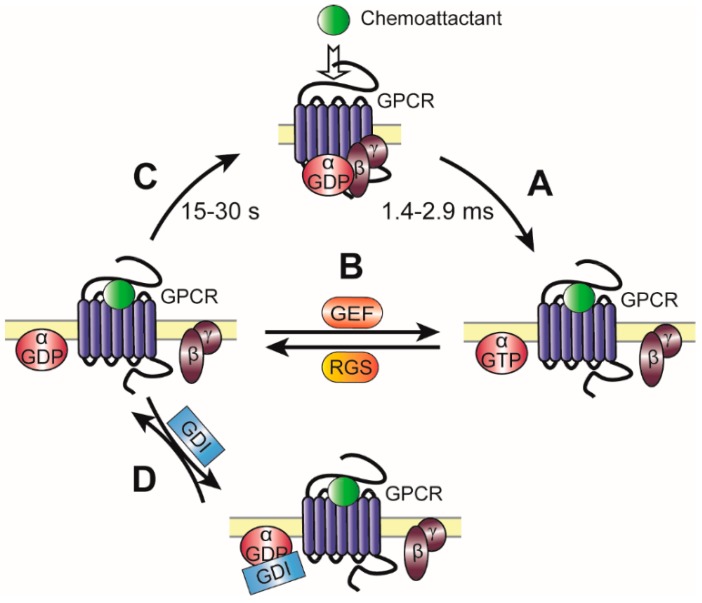
A schematic representation of mammalian Gα regulation. Upon binding of extracellular chemoattractant, GPCRs undergo conformational changes to act as guanine nucleotide exchange factors (GEFs) for Gα subunits, facilitating GDP release and subsequent binding of GTP, and release from Gβγ dimers (**A**) Non-receptor GEFs can bind to Gα-GDP and extend Gα subunit activation by stimulating the exchange of Gα-GDP to the active GTP-bound state. Regulator of G protein signaling (RGS) proteins stimulate the exchange of Gα-GTP back to Gα-GDP, serving as GTPase-accelerating proteins (GAPs) for Gα, thereby dramatically enhancing their intrinsic rate of GTP hydrolysis; (**B**) Upon GTP hydrolysis of Gα, the heterotrimer of Gα-GDP and Gβγ can reform, restoring the coupled GPCR/G protein complex; (**C**) However, in the presence of guanine nucleotide dissociation inhibitors (GDIs), Gα can become trapped in a Gα·GDP/GDI complex, preventing Gβγ from reassociation and re-coupling to GPCRs (**D**).

The general paradigm of GPCR activation is that ligand binding induces a conformational change in intracellular receptor domains resulting in the release of GDP from the Gα subunit (Figure 2). The GDP is quickly replaced by GTP from the cytosol, which promotes disassociation of the three subunits as Gα-GTP and a Gβγ dimer, both of which can regulate a diverse set of downstream effectors. Due to the intrinsic Gα-associated GTPase activity, GTP is hydrolysed to GDP, and the inactive heterotrimeric complex is re-associated [32]. The reassembled heterotrimeric G protein complex can form a complex with the GPCR again. However, it is not yet clear whether heterotrimeric G proteins only bind to activated receptors encountered upon lateral diffusion (collision coupling model) [8,33], or whether the G proteins are able to bind to GPCRs prior to activation (pre-coupled model) [34].

The kinetics of heterotrimeric G protein dissociation in response to chemoattractant have been extensively studied in both mammalian and *Dictyostelium* cells. Activation of the receptor occurs in the time frame of ms [35,36], with maximum dissociation of the heterotrimeric G protein complex within 3–6 s after uniform stimulation with chemoattractant [37]. The amount of dissociated Gα and Gβγ at the front and back of *Dictyostelium* cells corresponds to the relative amount of cAMP at the front and back of the cell, indicating that signal amplification occurs downstream of Gα and Gβγ proteins [38]. The rate limiting step in the heterotrimeric G protein activation cycle is re-association of Gα-GDP and Gβγ with the receptor, which can take up to 15–30 s in mammalian cells [37,39] and several minutes in *Dictyostelium* cells [40]. Because of the fast intracellular response upon receptor activation and slow re-association rate, the signaling rate of chemotaxis receptors is limited. Under continuous uniform stimulation, the downstream chemotaxis pathways, such as PIP3 production, adapt and *Dictyostelium* cells stop migrating, however, surprisingly, under these conditions, the heterotrimeric G proteins remain dissociated from the receptor. This strongly suggests that ligand-bound active receptors continuously activate Gα and Gβγ subunits, and that adaptation to the signal at least partly occurs downstream of heterotrimeric G proteins [40].

Based on the conventional heterotrimeric G protein cycle, the duration of downstream signaling is controlled by the lifetime of the Gα subunit in its GTP-bound state. In the last couple of years, several guanine nucleotide exchange factors (GEFs), GTPase-activating proteins (GAPs), guanine nucleotide dissociation inhibitor (GDIs) and regulators of Gβγ signaling have been identified that regulate and fine-tune the heterotrimeric G protein signaling during chemotaxis (Figure 2, [41]).

#### 2.2.1. Regulation of Gα Signaling by GEFs

In the conventional model of G protein signaling, GPCRs are the GEFs for Gα proteins, stimulating the exchange of G protein bound GDP to GTP and inducing dissociation of free Gα-GTP and Gβγ. However, in the last decades, several non-receptor GEFs have been identified that stabilize a nucleotide-free transition state of Gα, thereby reducing the high nucleotide affinity by many orders and promoting nucleotide release. This subsequently facilitates binding of GTP, which is present in excess over GDP in the cytosol of the cell. So far, several non-receptor GEFs have been identified that play an important role in regulating Gα activation during chemotaxis (Table 2). For instance, GIV (Gα-interacting vesicle-associated protein, also known as Girdin) has been described as a GEF for mammalian Gα_i3_ [42]. Through the GEF motif located in the C-terminus, GIV binds and exchanges Gα_i3_-GDP to Gα_i3_-GTP that is available for the activation of downstream effectors [42]. It has been revealed that GIV is required to stimulate the Gβγ-dependent PI3K/Akt pathway via GIV/Gα_i3_ activation, which remodels the actin cytoskeleton and regulates cell migration during cancer cell invasion [42,43,44,45]. 

**Table 2:** Regulation of Gα subunit signaling in chemotaxis.

Classification	G Protein Selectivity	Chemotactic Downstream Pathway
**GEF**		
GIV	Gα_i3_	PI3K/Akt pathway
Mammalian Ric-8A	Gα_i/o_, Gα_q_, and Gα_12_	Gα_q_-linked ERK activation
Mammalian Ric-8B	Gα_s_ and Gα_q_	Not defined
*D. discoideum* Ric8	Gα2and Gα4	Ras, small G proteins
**RGS**		
Mammalian RGS1	Gα_i_	Down-regulation of Gβγ
Mammalian RGS3	Gα_i_	Blocking binding of Gα to adenylyl cyclase
Mammalian RGS4	Gα_i_	MAPK pathways: ERK1/2 and p38MAPKs
Mammalian RGS13	Gα_i_ and Gα_q_	Intracellular calcium production and pERK1/2 induction
*D. discoideum* RCK1	Gα2	Not defined
**GDI**		
Mammalian AGS3/LGN	Gα_i_	Binding to Gαi-GDP and mInsc
Mammalian Rap1GAP	Gα_z_	Rap1/B-Raf/ERK pathway

Resistance to Inhibitors of Cholinesterase (Ric8A and Ric8B) are regulators of heterotrimeric G protein signaling that can act both as non-receptor GEFs and as chaperone for Gα proteins [46]. Ric-8A only interacts with GDP-bound Gα in the absence of Gβγ, resulting in release of GDP and formation of a stable, nucleotide-free Gα·Ric-8A complex. GTP then binds to Gα and disrupts the complex, releasing Ric-8A and the activated Gα protein [47]. Ric-8A is crucial for cranial neural crest cell migration; it localizes to the plasma membrane of the leading edge, where it amplifies Gα signaling to downstream effectors [48]. Furthermore, silencing of Ric-8A in embryonic fibroblasts inhibited PDGF-induced cell migration and prevented the translocation of Gα_13_ to the cell cortex [49]. Our work has shown that *Dictyostelium* Ric8 also serves as a non-receptor GEF that is important for development and chemotaxis to cAMP and folate [50]. *Dictyostelium* Ric8 is not important for the initial activation of Gα but competes with Gβγ to bind free Gα-GDP, and converts it back to the active Gα-GTP form. It thereby amplifies and extends the G protein signal. In contrast to mammalian Ric8, there is so far no evidence that *Dictyostelium* Ric8 in addition has a role as a chaperone for Gα proteins [51]. Both in mammals and in *Dictyostelium*, the regulation of Ric8 is still not completely understood. However, it has been shown that, in humans, RGS14 integrates conventional Gα_i1_ and Ric8A signaling, suggesting the presence of a heterotrimeric G protein regulator complex that contains both GAP and GEF activity [52].

#### 2.2.2. Regulation of Gα Signaling by RGS

The heterotrimeric G protein signal is terminated by hydrolysis of Gα-bound GTP by the intrinsic GAP activity of Gα subunits assisted by RGS proteins (Regulators of G protein Signaling) [53,54,55]. So far, more than 30 family members have been recognized that all contain a conserved RGS domain of approximately 130 amino acid residues in length which interact with active Gα subunits [56]. RGS proteins can regulate Gα signaling pathways in three ways: (i) they act as GAPs for Gα by stimulating the low intrinsic GTPase activity [57]; (ii) they act as effector antagonists that inhibit G proteins from binding to their effectors [58]; and (iii) they enhance the affinity of Gα subunits for Gβγ subunits after GTP hydrolysis, thereby accelerating reformation of the inactive heterotrimeric complex [57].

Several RGS proteins have been identified that play an important role in the regulation of chemotaxis (Table 2). The *Dictyostelium* RGS domain-containing protein kinase 1 (RCK1) has been described as a negative regulator of *Dictyostelium* chemotaxis, however the mechanism remains to be determined [59]. Human RGS1 and RGS3 are important for chemotaxis of germinal center B cells towards lymphoid tissue chemokines [37,39]. Overexpression of RGS1 and RGS3 in B cells impaired the recruitment of B cells to inflammatory sites initiated by the two chemoattractants, lysophosphatidic acid (LPA) and platelet-activating factor (PAF). RGS1 accelerates heterotrimeric complex formation [60], while RGS3 probably acts as an antagonist, blocking the binding of Gα to its effector adenylyl cyclase [39]. Human RGS4 acts as GAP for Gα_i_ which is important for the migration of Mv1Lu cells to fibronectin [61]. The constitutive expression of RGS13, a GAP for Gα_q_, reduces B cells chemotaxis to a variety of chemoattractants, including CXC chemokine ligand 12 (CXCL12), CXCL13, and CC chemokine ligand 19 (CCL19) [62]. Reversely, the reduction of RGS13 expression enhances chemoattractant signaling [62]. Consistently, RGS1/RGS13 double knock-out cells have a more polarized cellular morphology and improved chemotaxis towards CXCL12 [62]. Interestingly, Gβγ is also important in Gα signal termination by recruitment of the R7 family of RGS proteins (RGS6, 7, 9 and 11), and subsequent R7-Gβ_5_ dimerization which increases the GTPase activity of Gα subunits of the Gα_i_ family [63].

#### 2.2.3. Regulation of Gα Signaling by GDIs

Guanine nucleotide dissociation inhibitors (GDIs) are the third class of regulators of the heterotrimeric G protein cycle. Gα specific GDIs possess one or more highly conserved 19-amino acid polypeptide GoLoco (“Gα_i/o_-Loco” interaction) motifs that specifically interact and inhibit the nucleotide exchange of Gα proteins [64,65]. The GoLoco motif has been identified in several diverse proteins, including mammalian RGS12 and RGS14, Purkinje cell protein-2, Rap1GAP and GPSM2/LGN (Table 2, [65]). 

Mammalian Rap1GAP possesses both a Rap1-specific GTPase-activating protein domain and a GoLoco motif. Previously it was shown that Rap1GAP binds Gα_z_ in its GTP-bound state [66]. Gα_z_-mediated recruitment of Rap1GAP attenuated the Rap1-mediated Rap1/B-Raf/ERK signal transduction cascade, which stimulates cell migration [67]. 

Activator of G protein signaling 3 (AGS3) is another well characterized GDI for Gα_i_ [68]. AGS3 together with its homolog LGN translocates to the leading edge to form a Gα_i_-GDP·AGS3/LGN complex. This complex simultaneously binds to mInsc, which results in mInsc-mediated targeting of the Par3-Par6-aPKC complex to pseudopods at the leading edge to regulate directionality during neutrophil chemotaxis [69].

#### 2.2.4. Regulation of Gβγ Signaling

In addition, signaling of Gβγ is tightly regulated by various mechanisms. Phosducin has both been reported as an inhibitor and activator of Gβγ signaling [70]. Initial reports showed that phosducin competes with downstream effectors and Gα for binding to free Gβγ and thereby thus down regulates the chemotaxis response [71]. In contrast, recently phosducin-like protein 1 (PhLP1) was shown to enhance Gβγ signaling by acting as a cochaperone assisting in the assembly of Gβ and Gγ into a functional Gβγ complex [72]. Consistently, *Dictyostelium* cells lacking *phlp1* have strongly impaired heterotrimeric G protein signaling and are unable to chemotax [73,74]. 

Another regulator of Gβγ is a receptor of activated C kinase 1 (RACK1), which inhibits interaction between Gβγ and the downstream effectors PLCβ and PI3Kγ, therefore overexpression of RACK1 or RACK1 fragments leads to decreased leukocyte chemotaxis [75]. Conversely, WD40-repeat containing protein 26 (WDR26) might act as a positive regulator of Gβγ signaling functioning as a scaffold that recruits and translocates Gβγ effectors, suppression of WDR26 in HL60 cells resulted in loss of directionality and cell migration speed. Moreover, WDR26 suppression blocks RACK1 interaction with Gβγ, indicating that RACK1 functions downstream of WDR26 [76].

### 2.3. Heterotrimeric G Protein Activated Chemotaxis Pathways

**Figure 3:**
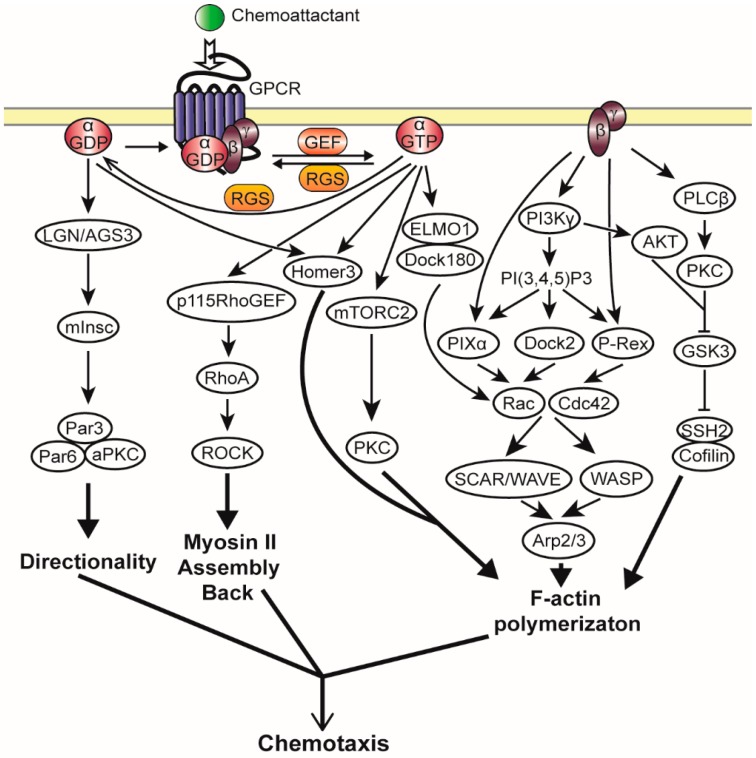
A schematic representation of the chemotactic signaling pathways in mammalian neutrophils. In the presence of PIP3, Gβγ can directly activate GEFs for Rac and Cdc42, resulting in activated Rac and Cdc42 to promote F-actin polymerization and regulate cell motility of migrating neutrophils through the activation of WASP and SCAR/WAVE complex. During chemotaxis, several downstream effectors of Gα subunits have been identified: ELMO1/Dock180, p115RhoGEF, mTORC2, Homer-3 and LGN/AGS3-mInsc.

As described above, amplification and cell polarization during chemotaxis is established downstream of receptor binding and heterotrimeric G protein activation. Recent studies have identified a complex network of GPCR regulated interconnecting signaling pathways that lead to the intracellular amplification of the extracellular chemoattractant gradient that includes the preferential activation of monomeric G proteins at the leading edge, major changes in the cytoskeleton with actin polymerization at the leading edge, and actin-myosin filament assembly at the rear and sides of the cell [3]. The new actin filaments induce the formation of local pseudopodia, while the acto-myosin filaments inhibit pseudopod formation in the rear and retract the uropod; in this way, coordinated cell movement is achieved [9]. Many chemotaxis pathways that are directly regulated by Gβγ have been identified (Figure 3, [77,78]); however, we are only beginning to understand whether Gα-GDP/GTP exchange mediates downstream signaling mainly through the release of Gβγ and/or whether distinct signaling pathways are regulated through Gα-GTP and/or Gα-GDP subunits.

#### 2.3.1. Gβγ Mediated Chemotaxis Pathways

Many studies have suggested that the Gβγ dimer functions as the main transducer of chemotactic signals [79]. Deletion of the single *Gβ* gene in *Dictyostelium* completely impairs the chemotactic response [80]. In *Dictyostelium*, the released Gβγ interacts with PI3K in a Ras-dependent manner [81]. The product of PI3K, PtdIns(3,4,5)P3 (PIP3), then accumulates asymmetrically at the leading edge of chemotaxing cells and provides a binding site for a subset of PH-domain-containing proteins, including cytosolic regulator of adenylyl cyclase (CRAC) protein and PKB/Akt (protein kinase B). CRAC acts as a stimulator for the adenylyl cyclase (ACA), which leads to the production of cAMP that is rapidly secreted to stimulate neighboring cells, a process that is essential for *Dictyostelium* development [82,83]. PKB/Akt contributes to the regulation of cell polarity and chemotaxis through the activation of PAKa, which is required for myosin II assembly at the rear of chemotaxing cells [84]. In addition PIP3 activates Rac small G proteins through Rac specific GEFs, resulting in the regulation of Wiskott–Aldrich syndrome protein (WASP) and the SCAR/WAVE complex [85], which stimulate the Arp2/3 complex that is required for the production of branched filaments and pseudopod extension [86]. Moreover, a PH domain-containing protein A (PhdA) is another PI3K interacting PH-domain-containing protein that regulates actin polymerization and cell polarization [87]. 

Despite many efforts, so far, only ElmoE has been identified as direct Gβγ effector in *Dictyostelium* [88]. Gβγ mediated activation of ElmoE promotes actin polymerization at the leading edge of chemotaxing *Dictyostelium* cells via the association of Dock-like proteins that function as activator for small G proteins of the Rac family [88]. In mammalian neutrophils, in the presence of PIP3, Gβγ can directly bind and activate P-Rex1 [89] and PIXα [90], which are GEFs for Rac and Cdc42, respectively. This results in a gradient of activated Rac and Cdc42 to activate the WASP and SCAR/WAVE complex at the leading edge, which is important for the regulation of cell motility and migration of neutrophils [59,63]. Additionally, Dock2, another PIP_3_-dependent GEF for Rac, has been described to play a predominant role in regulating leading edge formation [91,92]. Activated Rac and Cdc42 together with polymerized actin act as a positive feedback loop that recruits more PIP_3_ in the leading edge of the cell; however the mechanism of how PIP_3_ is recruited still remains unclear [93]. A recent study reveals that Gβγ can directly activate phospholipase PLCβ and its effector PKC (protein kinase C) [94]. Further work demonstrates that both PLCβ-PKC and PI3Kγ-Akt signaling pathways can phosphorylate and deactivate Glycogen synthase kinase 3 (GSK3) that attenuates GSK3-mediated inhibition of the cofilin phosphatase SSH2 (slingshot2) and dephosphorylation of cofilin, leading to fMLP-induced neutrophil polarization and actin cytoskeleton remodeling [94].

#### 2.3.2. Gα Mediated Chemotaxis Pathways

For a long time, Gα subunits were only considered as a “timer” to govern Gβγ signaling by regulating the release and reassociation of the Gβγ dimer. However, recently the first mammalian Gα effectors important for chemotaxis have been reported (Figure 3, [69]), including Homer3 that we identified as a novel Gα_i2_-binding protein that spatially organizes actin assembly to support polarity and motility during neutrophil chemotaxis [95]. Gα_i2_ also interacts with the Elmo1/Dock180 complex functioning as a RacGEF to activate Rac1 and Rac2 proteins, thereby inducing actin polymerization and cell migration [96]. Additionally, mInsc indirectly binds Gα_i2_-GDP via LGN/AGS3 and helps maintain directionality in neutrophils through its downstream effector of Par3-Par6-aPKC complex [69]. Furthermore, the Gα_12_-mediated mTORC2 (mammalian TORC2)-PKC signaling pathway is critically important for LPA induced fibroblast migration [97]. Interestingly, via the Rho specific GEF, p115RhoGEF, Gα_12/13_ is able to activate Rho and its effector kinase ROCK, leading to myosin II assembly at the rear of chemotaxing cells [98,99]. Gα-mediated pathways thus seem to be important for both the signaling at the leading edge and rear of the cell. Surprisingly, the ELMO1/Dock180 complex interacts specifically with Gα_i2_-GTP [99], while LGN/AGS3 specifically interacts with Gα_i2_-GDP at the leading edge of migrating neutrophils [69]. This might suggest that free Gα can activate downstream pathways independent of their nucleotide bound state.

## 3. Conclusions

Tight regulation of GPCR and heterotrimeric G protein signaling on several levels is essential for proper chemotaxis. Receptor affinity and expression is strictly regulated, thereby increasing the range of concentrations to which cells can respond. Recent studies have shown the importance of non-canonical regulators for chemotaxis: RGS, GDI and GEF proteins either extend or shorten the lifetime of Gα-GTP, thereby fine-tuning the chemotactic response. Accumulating evidence suggests that both Gα and Gβγ have their separate downstream pathways; however, which nucleotide bound state of Gα transduces the signals and the exact link between heterotrimeric G protein activation and cytoskeletal rearrangements remains to be determined. It is of high interest to fully understand the regulations and intracellular targets of chemotaxis receptors, which could help in finding new drug targets for several diseases, including cancer, asthma and rheumatoid arthritis.
